# A new unconventional HLA-A2-restricted epitope from HBV core protein elicits antiviral cytotoxic T lymphocytes

**DOI:** 10.1007/s13238-014-0041-4

**Published:** 2014-03-22

**Authors:** Lu Sun, Yu Zhang, Bao Zhao, Mengmeng Deng, Jun Liu, Xin Li, Junwei Hou, Mingming Gui, Shuijun Zhang, Xiaodong Li, George F. Gao, Songdong Meng

**Affiliations:** 1 CAS Key Laboratory of Pathogenic Microbiology and Immunology, Institute of Microbiology, Chinese Academy of Sciences (CAS), Beijing, 100101 China; 2 Beijing Institute of Infectious Diseases, Beijing 302 Hospital, Beijing, 100039 China; 3 Xinjiang Agricultural University, Ürümqi, 830052 China

**Keywords:** chronic hepatitis B, HLA-A2, HBc peptide, CTL response, antiviral cytotoxity

## Abstract

Cytotoxic T cells (CTLs) play a key role in the control of Hepatitis B virus (HBV) infection and viral clearance. However, most of identified CTL epitopes are derived from HBV of genotypes A and D, and few have been defined in virus of genotypes B and C which are more prevalent in Asia. As HBV core protein (HBc) is the most conservative and immunogenic component, in this study we used an overlapping 9-mer peptide pool covering HBc to screen and identify specific CTL epitopes. An unconventional HLA-A2-restricted epitope HBc141–149 was discovered and structurally characterized by crystallization analysis. The immunogenicity and anti-HBV activity were further determined in HBV and HLA-A2 transgenic mice. Finally, we show that mutations in HBc141–149 epitope are associated with viral parameters and disease progression in HBV infected patients. Our data therefore provide insights into the structure characteristics of this unconventional epitope binding to MHC-I molecules, as well as epitope specific CTL activity that orchestrate T cell response and immune evasion in HBV infected patients.

## Introduction

Around 350 million people worldwide are chronically infected with Hepatitis B virus (HBV). Chronic HBV infection is a major cause of cirrhosis, liver failure, and hepatocellular carcinoma (HCC) (Lavanchy, 2005; Neuveut et al., 2010). HBV-specific CD8^+^ T lymphocytes (CTL)-mediated immune response is multi-specific, polyclonal, and vigorous during acute hepatitis B (AHB), which plays a vital role in viral control and viral clearance, as well as disease pathogenesis (Yukihiro, 2012; Westover and Hughes, 2007; Tan et al., 2008). Whereas HBV-specific CTL response is minimal or undetectable in chronic hepatitis B (CHB) with viral persistence and immune tolerance, indicating the key role of HBV-specific T-cell response in determination of disease progression and outcome (Bertoletti and Gehring, 2006; Zhang et al., 2013).

The HBV genome of ~3.2 kb in length efficiently encodes several overlapping viral proteins, including the polymerase, core, HBe, envelope (Pre-S1, S2, S), and X proteins. Analysis of CTLs specific for viral epitopes within core (Sendi et al., 2009; Liu et al., 2012;), envelope (Liu et al., 2008), polymerase (Rehermann et al., 1995), and X (Hwang et al., 2002) proteins showed that the highly conserved HBV core protein (HBc) elicits the strongest CTL responses than other viral proteins, suggesting that HBc-specific T cell response may play a leading role in viral control and clearance. To identify immune-dominant HBV-specific CTL epitopes, especially epitopes from HBc protein, is therefore necessary for monitoring T cell responses during disease progression, as well as for developing epitope-based therapeutic vaccines against CHB (Inchauspe and Michel, 2007; Gordon et al., 2013; Liu et al., 2013a, b).

So far 60 HBV-specific human leukocyte antigen (HLA) class I restricted and 32 HBV-specific HLA class II restricted epitopes have been identified in all 8 HBV genotypes (Desmond et al., 2008; Liu et al., 2008; Guo et al., 2011; Zhang et al., 2013; Chen et al., 2013; Tan et al., 2013). However, most of these identified epitopes are derived from HBV of genotypes A and D, few HLA class I restricted epitopes have been defined in virus of genotypes B and C, which are more prevalent in Asia. Meanwhile, currently the mostly used methods to predict and identify T cell epitopes are either by computer algorithms based on the mode of peptide binding to MHC molecules, or measurement of T cell responses of PBMCs simulated with panels of overlapping peptides (Liu et al., 2011). These epitope identification strategies may ignore unconventional T cell epitopes as computational analysis is largely based on the characteristic of anchor residues.

In this study, an overlapping 9-mer peptide pool was used to screen and identify HBV genotypes B- and C-derived T cell epitopes. A new unconventional CD8^+^ T cell epitope HBc141–149 derived from viral core protein, which shares partial sequence identity with previously reported HBc141–151 (Bertoni et al., 1997) and HBc139–148 (Lee et al., 1997), was discovered. Its immunological function and clinical relevance were further assessed.

## Results

### Identification of a new HLA-A*0201-restricted epitope from HBV core protein

**Figure 1:**
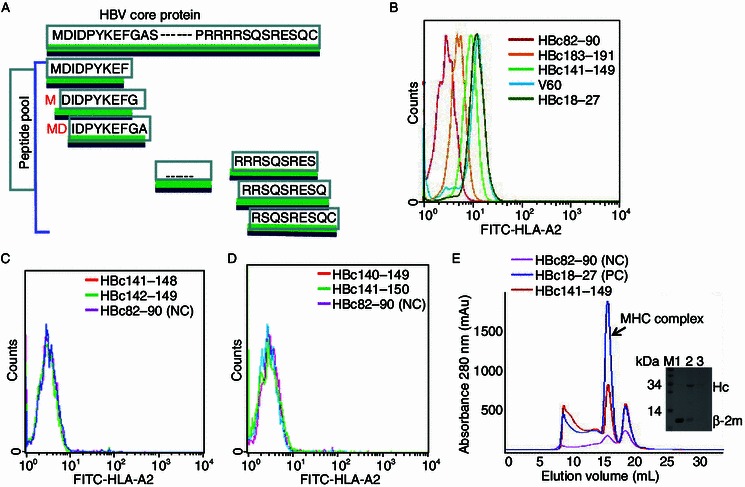
Binding affinity of HBc141–149 peptide to HLA-A*0201. (A) The overlapping method of 8 aa overlap was used to synthesize a total of 191 nonapeptides covering HBc aa 1–150. (B) MHC stabilization assay with T2 cells was used to quantify peptide binding affinity of HBc18–27, HBc82–90, HBc183–191, HBc141–149, and HBc60–68 (V60) to HLA-A*0201 molecules by flow cytometry, respectively. HBc18–27 and HBc82–90 peptides served as positive and negative controls, respectively. The results shown are representative of three independent experiments. (C and D) Measurement of the binding affinity of HBc141–149N- or C-terminally truncated (C) or extended (D) peptides to HLA-A*0201. HBc82-90 served as a negative control (NC). (E) *In vitro* refolding of HBC141–149 peptide with HLA-A*0201 heavy chain and β2m. Gel filtration chromatography was used to analyze the refolded complexes on a Superdex200 16/60 column. The HLA complex with the expected molecular mass of 45 kDa eluted at the volume of 15.9 mL. The HLA complex (peak 2) was analyzed by SDS-PAGE electrophoresis and coomassie blue staining (line 2)

HBV core protein is the most conservative and immunogenic component of HBV proteins. We used an overlapping 9-mer peptide pool covering the whole length of core protein (aa 1–150) and its variants (totally 191 peptides) for T2 binding assay to screen genotype B- and C virus-derived HBcAg-specific T cell epitopes (Zhang et al., 2013), as shown in Fig. 1A. Several peptides were found to have high affinity for binding to HLA-A*0201 molecules, as evidenced by the FI (0.72 for HBc183–191, 2.41 for HBc141–149, 2.64 for HBc60–68 (V60), 2.47 for HBc18–27, and 0.04 for HBc82–90), as shown in Fig. 1B. HBc141–149 spanning from HBc aa 141 to 149 was chosen as the focus of this study due to its high binding affinity among the top hits from screening. To determine the minimal sequence length of the epitope, panels of N- or C-terminally truncated or extended peptides of HBc141–149 were synthesized. The FIs of truncated peptide HBc141–148 (STLPETTV) and HBc142–149 (TLPETTVV) were only 0.124 and 0.151, respectively (Fig. 1C). The FIs of decapeptides HBc140–149 (LSTLPETTVV) and HBc141–150 (STLPETTVVR) were only 0.033 and 0.005, respectively (Fig. 1D). All HBc141–149 truncated and extended peptides displayed little binding to HLA-A*0201, which indicates that the 9-mer peptide HBc141–149 is the optimized epitope in length. In addition to T2 cell binding assay, the capability of HBc141–149 to bind to HLA-A*0201 was observed in the refolding assay (Fig. 1E).

### The structure of HLA-A*0201/HBc141–149 complex

**Figure 2:**
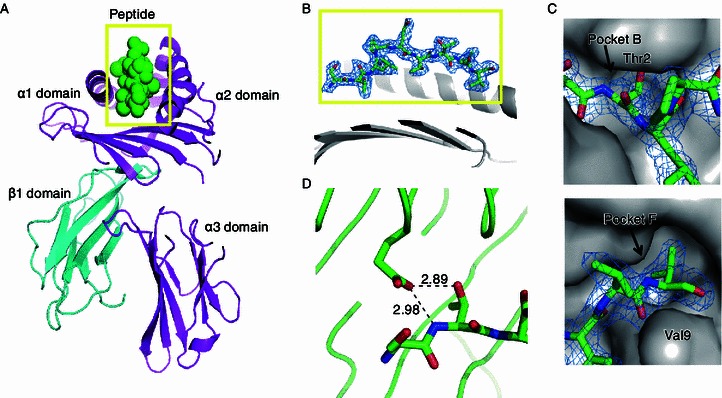
Structure of HLA-A*0201 complexed with HBc141–149. (A) Overview of the structure of HBc141–149 peptide presented by HLA-A*0201; HBc141–149 is presented in the peptide-binding cleft. (B) HBc141–149 peptide is presented in the peptide-binding clefts with electron density at the 1σ contour level. (C) Residues Thr2 and Val9 (green sticks) are located in pocket B and pocket F of HLA-A*0201. The heavy chain of HLA-A*0201 is show in gray surfaces. (D) The side chain OH of Thr forms hydrogen bond interaction with H atom of Glu on the alpha 1 domain of the heavy chain

Next, the complex of HLA-A2 and HBc141–149 was prepared for crystallization to characterize the binding features of HBc141–149. The crystal structure of the HLA complex was determined to 2.3 Å resolution (Table 1). The structure of HLA-A*0201/HBc141–149 shows that HBc141–149 possesses a typical conformation of an HLA-A2-restricted 9-mer epitope (Fig. 2A and 2B). The unambiguous electron densities of HBc141–149 clearly show that position 2 (T2) and position 9 (V9) are buried in pockets B and F, respectively (Fig. 2C). Compared to the typical HLA-A2-restricted epitopes which have an anchor residue Leu or Met at position 2, HBc141–149 has Thr at position 2. The side chain OH of Thr does not disrupt its inserting into the hydrophobic pocket B properly (Fig. 2D). Instead, the side chain OH of Thr can form a strong hydrogen bond interaction with H atom of Glu on the α1 domain of the heavy chain, which helps HBc141–149 binding to the HLA-A2 heavy chain and stabilizes the entire complex. The side chains of amino acids P4, E5, and V8 protrude out from the HLA-A2 surface and may be involved in T cell receptor (TCR) attachment and recognition. To the best of our knowledge, this is the first structure showing the binding features of HLA-A2 to HBc141–149 with an unconventional P2 anchor Thr.

**Table 1:** X-ray diffraction data processing and refinement statistics

	HLA-A2/HBc141–149
**Data processing**	
Space group	P2_**1**_
Cell parameters (Å)	
a (Å)	52.980
b (Å)	80.787
c (Å)	56.488
α (°)	90.000
β (°)	112.596
γ (°)	90.000
Resolution range (Å)	50.00–2.30
Total reflections	96418
Unique reflections	19518
Completeness (%)	99.8 (99.2)
Redundancy	4.9 (4.3)
*R*_merge_ (%)	0.123 (0.435)
*I*/*σ*	12.868 (2.939)
**Refinement**	
*R*_work_ (%)	0.2142
*R*_free_ (%)	0.2651
r.m.s. deviation	
Bond lengths (Å)	0.002
Bond angles (°)	0.627

Values in parentheses are given for the highest resolution shell

### HBc141–149 peptide generates specific CTL response in HLA-A2.1/Kb transgenic mice

**Figure 3:**
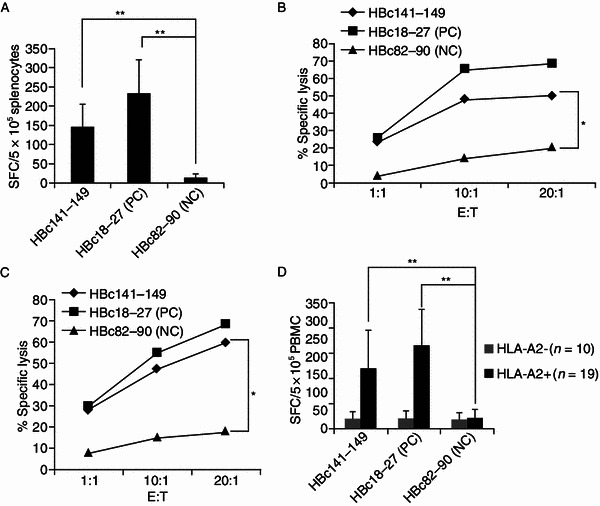
**Detection of HBc141–149 peptide-specific CD8**^**+**^**T cell response in HLA-A2.1/Kb mice and AHB patients**. Female HLA-A2.1/Kb transgenic mice were immunized with an HBV DNA prime/peptide boost regimen at weeks 1, 3, and 4. HBc82–90 and HBc18–27 peptides served as negative and positive controls in immunization, respectively. (A) Fresh splenocytes (5 × 10^5^) isolated from immunized mice were stimulated with HBc141–149, HBc18–27 or HBc82–90 peptide, and peptide-specific CTLs were detected by IFN-γ ELISPOT assay. (B and C) 293T cells labeled with CFSE were transfected with pHBV1.3 as target cells (B), and T2 cells labeled with CFSE were loaded with 20 μg/mL peptide at 37°C for 1 h as target cells (C). The target cells were then mixed with HBc141–149-stimulated splenocytes at different ratios: 1:1, 1:10, and 1:20, or HBc18–27, HBc82–90-stimulated splenocytes served as positive or negative controls. After 4 h, the mixed samples were stained with PI, and the killing of target cells were analyzed by FACS. The data shown are the mean ± SD of five mice. (D) AHB patients (*n* = 29) were divided into HLA-A2-positive (*n* = 19) and HLA-A2-negative (*n* = 10) groups. PBMCs (2 × 10^5^/well) from these patients were stimulated with the indicated peptides for detection of peptide-specific CTLs by ELISPOT assay. HBc82–90 peptide served as negative control for background evaluation in ELISPOT assay. PBMCs from HLA-A2- patients were used for specificity evaluation of CTLs. Paired samples *t*-tests were used for ELISPOT assay in AHB patients. *P < 0.05 and **P < 0.01 by *t*-test. Data are representative of two independent experiments

Then, the immunogenicity of HBc141–149 epitope was determined *in vivo*. Female HLA-A2.1/Kb transgenic mice were immunized with HBc DNA-prime/HBc141–149 peptide boost regimen three times, using heat shock protein gp96 as adjuvant (Li et al., 2011). HBc141–149-specific CTL was detected by ELISPOT assay 1 week after the last immunization. As can be seen in Fig. 3A, similar to the HBc18–27 peptide-immunized mice (positive control), a strong CTL response was observed in splenocytes from HBc141–149 peptide-immunized mice (SFC, 145.4 ± 58.6). No peptide-specific CD8^+^ T cell response was detected from HBc82–90 peptide-immunized mice (negative control). Similar results were observed in the killing assay using HBV plasmid-transfected 293T cells (Fig. 3B) or HBc141–149 peptide-pulsed T2 cells (Fig. 3C) as target cells.

### HBc141–149 is a naturally processed epitope in AHB

To further determine the epitope-specific CTLs, fresh PBMCs from HLA-A2^+^ AHB patients were stimulated with HBc141–149 peptide and detected by *ex vivo* IFN-γ ELISPOT assays. As shown in Fig. 3D, a much higher peptide-specific CTL response was observed in PBMCs stimulated with HBc141–149 or the immunodominant peptide HBc18–27 as the positive control than negative control peptide HBc82–90 (119.68 ± 76.66 for HBc141–149 or 164.58 ± 112.41 for HBc18–27 vs. 20.94 ± 17.57 for HBc82–90, both *P* < 0.01). The high standard deviations observed in ELISPOT assay may reflect the random between-patient variation. Taken together, these results indicate that HBc141–149 is an HLA-A2-restricted CD8^+^ T cell epitope and is naturally processed in patients with HBV infection.

### HBc141–149 epitope elicits antiviral T cell immunity in HLA-A2.1/HBV transgenic mice

**Figure 4:**
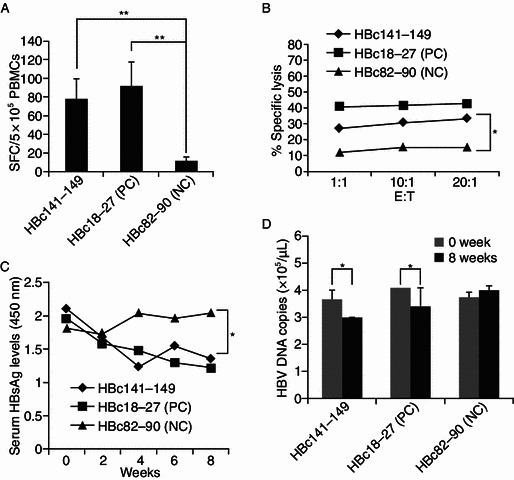
**Immunization with HBc141–149 peptide induces specific CTL responses inhibits viral replication in HLA-A2**^**+**^**/HBV transgenic mice**. F1 hybrids of HLA-A2.1/kb transgenic mice and HBV transgenic BALB/c mice (*n* = 5 in each group) were immunized three times as in Fig. 3. (A) ELISPOT assay. Splenocytes from immunized mice were stimulated with HBc141–149 peptide, or with HBc82–90 or HBc18–27 as negative or positive controls. (B) 293T cells labeled with CFSE were transfected with pHBV1.3 as target cells, and mixed with HBc141–149-stimulated splenocytes at different ratios: 1:1, 1:10, and 1:20, or with HBc18–27 or HBc82–90-stimulated splenocytes served as positive or negative controls . After 4 h, the mixed samples were stained with PI and analyzed by FACS. (C) Serum secretion of HBsAg was detected by ELISA every other week. (D) HBV DNA levels in serum were quantified by real-time PCR at weeks 0 and 8. The data shown are the mean ± SD of five mice. Paired *t*-tests were used. *P < 0.05 and **P < 0.01. Data are representative of two independent experiments

Next, we examined whether HBc141–149 epitope was able to induce anti-HBV T cell response using F1 hybrids of HBV transgenic BALB/C mice and HLA-A2.1/kb transgenic mice as the experimental model, which are HBV immunotolerant. HLA-A2.1/HBV transgenic mice were immunized with an HBc DNA prime/ HBc141–149 peptide boost formulation. As shown in Fig. 4A, compared to mice immunized with negative control peptide, the number of SFCs increased by around 6-fold in HBc141–149 peptide-immunized mice. Similar result was obtained in cytotoxicity assay using HBV plasmid-transfected 293T cells as target cells. We then evaluated HBc141–149 peptide-induced T cell response could lead to inhibition of HBV. Immunization with HBc141–149 led to a 35.5% decrease in serum HBsAg levels (*P* < 0.05) (Fig. 4C), and a significant reduction of viral DNA levels (*P* < 0.05) (Fig. 4D) at week 8 compared to the negative control. Meanwhile, significant lower levels of serum HBsAg and viral DNA were observed after immunization (at week 8) than those before immunization (at week 0) in HBc141–149-treated mice but not in control peptide-treated mice. And these results indicate that the epitope-specific CTL response induced by the HBc141–149 could significantly inhibit HBV replication in the transgenic mice.

### HBV accumulates mutations within HBc141–149 epitope in HBV-infected patients

**Figure 5:**
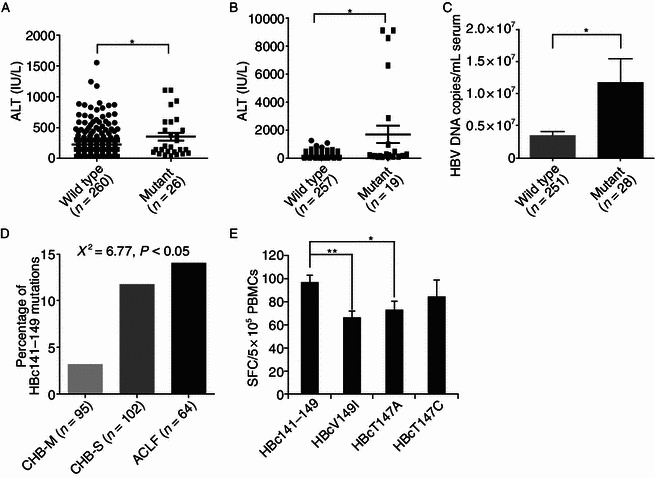
**The HBc141–149 mutations are correlated with ALT/AST levels, viral DNA loads and disease progression in HBV-infected patients**. (A–C) Distribution of serum ALT (A) and AST levels (B), and HBV DNA loads (C) in CHB patients infected with HBV isolates containing wild-type (STLPETTVV) or mutant HBc141–149. Student’s *t*-test was used to determine *P*-values. *P < 0.05. (D) Distribution of the HBc141–149 mutation rate in CHB and ACLF patients. CHB-M, mild chronic hepatitis B; CHB-S, severe chronic hepatitis B; ACLF, acute-on-chronic liver failure. Pearson’s χ test was used to determine *P*-values. (E) Female HLA-A2.1/Kb transgenic mice were immunized with the wild-type HBc141–149 or mutant (HBcV149I, HBcT147A or HBcT147C) peptide as in Fig. 3. Fresh splenocytes (5 × 10^5^) isolated from immunized mice were stimulated with wild-type or mutant peptide, respectively. Peptide-specific CTLs were detected by IFN-γ ELISPOT assay

Finally, to address the clinical relevance of the HBc141–149 epitope in CHB, mutations within this epitope were analyzed in 197 CHB and 64 ACLF patients (Table 2). In CHB, compared to patients infected with wild-type isolates (HBc141–149, STLPETTVV), patients infected with HBc141–149 mutants had much higher ALT levels (247.7 ± 18.62 vs. 545.2 ± 137.7, *P* < 0.05) (Fig. 5A) and aspartate aminotransferase (AST) levels (187.6 ± 13.78 vs. 1668 ± 700.5, *P* < 0.05) (Fig. 5B). Notably, compared to CHB patients infected with HBc141–149 wild-type viruses, patients with HBc141–149 mutants had much higher (approximately 3.3-fold) HBV DNA loads (*P* < 0.05) (Fig. 5C). There were no statistical differences in sex and age between patients infected with the wild-type and mutant isolates. The prevalence of the HBc141–149 mutations increased with the disease progression in HBV-infected patients (Fig. 5D). To investigate the impact of these sequence variations within HBc141–149 epitope on its immunogenicity, three 9-mer peptides containing main epitope variations of HBcV149I, HBcT147A, and HBcT147C in CHB patients were synthesized, respectively, for HLA-A2.1/Kb transgenic mice immunization. As seen in Fig. 5E, compared to the wild-type HBc141–149 peptide-immunized mice (SFC, 96.7 ± 6.02), CTL responses by ELISPOT assay were significantly decreased in HBcV149I mutant peptide- (SFC, 66.3 ± 5.69) or HBcT147A mutant peptide- immunized mice (SFC, 73 ± 7.81). Taken together, these results suggest that the HBc141–149 mutations associated with necroinflammation and higher HBV levels, and it may also be associated with poor prognosis, which may be due to viral immune evasion.

**Table 2:** Clinical and virologic characteristic of CHB-M, CHB-S, and ACLF patients

	CHB-M	CHB-S	ACLF	*P*-Value
Numbers	95	102	64	
Age (mean ± SD)	42.51 ± 13.7	43.37 ± 14.57	45.89 ± 12.09	*P* < 0.01
Gender (M/F)	12/83	18/84	13/42	*P* > 0.05
HBV genotype				
B (%)	17 (17.9%)	17 (16.7%)	5 (7.8%)	*P* > 0.05
C (%)	78 (82.1%)	85 (83.3%)	59 (92.2%)	*P* < 0.01
HBc141–149 variation	3 (3.1%)	12 (11.8%)	9 (14.1%)	*P* < 0.05
HBe positive (%)	72 (86.3%)	49 (47.1%)	6 (9.3%)	*P* < 0.01
HBV DNA copies/Ml (mean)	2.53 × 10^6^	8.26 × 10^6^	1.31 × 10^6^	*P* > 0.05
(range)	(2354–3.5 × 10^7^)	(500–2.64 × 10^8^)	(5980–1.32 × 10^7^)	
ALT(IU/L) (mean ± SD)	140.2 ± 186.7	284.5 ± 283.6	211.8 ± 244.1	*P* < 0.01
AST(IU/L) (mean ± SD)	87.7 ± 91.0	322.3 ± 932.2	238 ± 257.9	*P* < 0.01

HBV, hepatitis B virus; CHB-M, mild chronic hepatitis B; CHB-S, severe chronic hepatitis B; ACLF, acute-on-chronic liver failure; M, male; F, female; HBeAg, hepatitis B e antigen; ALT, alanine aminotransferase; AST, aspartate aminotransferase

## Discussion

In this study, we identified a new HLA-A2-restricted CD8^+^ T cell epitope HBc141–149 by screening an overlapping 9-mer peptide pool covering HBV core protein. This unconventional HLA-A2 restricted epitope was further determined and structurally characterized by HLA-A2 transgenic mouse model and crystallographic analysis. Moreover, we demonstrated that the HBc141–149 epitope exhibits antiviral capability in HLA-A2.1/HBV transgenic mice. Finally, our results show that mutations in HBc141–149 epitope correlate with clinically relevant parameters in CHB. Our work may therefore provide a comprehensive evaluation of the impact of the newly defined epitope on viral specific T cell response and suggests a possible immune evasion for maintenance of viral persistence in patients with HBV infection.

The identification of HBV-specific T cell epitopes is mostly based on the binding mode of the peptides to MHC molecules in silico prediction or assessing T cell responses with panels of overlapping peptides in HBV-infected patients. These methods are effective and rapid to purposefully identify epitopes, however, a considerable number of atypical or unconventional epitopes may be ignored or missed by these methods due to the limitation of anchor residues analysis-based computation (Liu et al., 2011). In this study, an overlapping 9-mer peptide pool was used to screen B and C genotype-derived HBc-specific CTL epitopes, and HBc141–149 was identified as a new 9-mer HLA-A2-restricted T cell epitope. Importantly, similar numbers of HBc141–149-specific and immunodominant epitope HBc18–27-specific CD8^+^ T cells were observed in AHB patients (Fig. 3C), indicating that HBc141–149 is an immunodominant epitope in HBV-infected patients. However, the newly defined epitope HBc141–149 (STLPETTVV) in this study possesses Thr at position 2. Different T cell epitope prediction programs, including SYFPEITHI (http://www.syfpeithi.de/Scripts/MHCServer.dll/Epitope-Prediction.htm), NETMHC (http://www.cbs.dtu.dk/services/NetMHC/), and BIMAS (http: //www-bimas.cit.nih.gov) show the binding score of the epitope HBc141–149 to HLA-A2 is only 0, 1.465, and 0.414, respectively. As HBc141–149 does not get a high score, it could be omitted in conventional screening. Interestingly, as for HBc141–149 (STLPETTVV), the side chain OH of Thr2 can form a stronger hydrogen bond interaction with Glu on α1 domain of the heavy chain, which helps its inserting into pocket B (Fig. 2D) of HLA-A*0201 and stabilize the entire complex. Therefore, our data indicated that Thr may also act as a dominant role as a P2 anchor for HLA-A2-binding peptides, which may be taken into consideration in the future designation of these online prediction programs.

CD8^+^ T cells are the main effector cells responsible for viral clearance as well as disease pathogenesis during HBV infection (Thimme et al., 2003; Harari et al., 2006; Ouyang et al., 2013). As a consequence of long-term interaction between HBV and infected patients, the virus evolves mutations to reduce epitopes for evading immune detection and clearance, especially escape from T cell recognition (Maman et al., 2011; Westover and Hughes, 2007). Indeed, nonsynonymous mutations in HBV epitope have been found to be associated with disease progression in CHB (Kim et al., 2011; Frelin et al., 2009). Consistent to these studies, we found that mutations in the newly defined epitope are positively related to viral parameters and pathogenesis of liver disease. The effect of mutations within HBc141–149 on viral replication capability, as well as the potential impact of the epitope-specific CTLs on the interplay of HBV and chronically infected patients remains to be addressed.

In summary, this study indicates that HBc141–149 is an immunodominant HLA-A2- restricted CTL epitope with atypical binding characteristics to HLA-A2 molecules, has potent anti-HBV immune activity and the clinical significance in patients with HBV infection. We further demonstrated that mutations within this epitope may affect disease progression in CHB. Given the key role of CD8^+^ T cells in viral clearance, our work provides valuable insight for the functional implications of HBc141–149 epitope-specific T cell response in HBV infection. Understanding the epitope-specific CTL function in the complex regulation networks that orchestrate T cell response, viral persistence and immunoevasion in CHB will allow to predict disease progression and develop immunotherapeutic approaches against HBV infection.

## Materials and methods

### Patients and blood samples

The database of HBV infected patients’ records built by the 302 Hospital (Beijing, China) (http://www.hbvdb.com) was used to perform the Clinical information analyses of HBc141–149. In this study we investigated the associations between HBc141–149 and the progress of liver disease or patients’ clinical indicators. The criteria for diagnosis of acute hepatitis B (AHB), chronic hepatitis B (CHB), acute on chronic liver failure (ACLF) complied with those of the Management Scheme of Diagnostic and Therapy of Viral Hepatitis and the Diagnostic and Treatment Guideline for Liver Failure, was issued by the Chinese Society of Infectious Diseases and Parasitology and the Chinese Society of Hepatology. CHB is further divided into two groups: mild chronic hepatitis B (CHB-M) and severe chronic hepatitis B (CHB-S). The criteria for CHB-M patients: mild-to-moderate liver disease activities which do not attain the criteria of CHB-S. The criteria for CHB-S patients: severe liver disease with evident clinical manifestation and significant alteration of biochemical parameters (serum albumin level ≤32 g/L, serum total bilirubin (TBIL) >85.5 μmol/L, plasma prothrombin activity (PTA) 40%–60%, or serum cholinesterase <4500 IU/L (Xu et al., 2011). The criteria for ACLF: a history of CHB with symptoms of extreme fatigue and severe digestive problems with biochemical parameters of TBIL >10 times normal level (171 μmol/L) and PTA ≤40% (Yang et al., 2002). All the enrolled patients’ clinical characteristics are described in Table 2.

A total of 29 AHB patients were enrolled for blood collection, which were divided into two groups: HLA-A2-positive (*n* = 19) and HLA-A2-negative (*n* = 10). All patients were negative for HCV, HDV, and HIV-1 infection. 10 mL of blood samples were collected from each patient. All patients were hospitalized in Beijing 302 Hospital from September 2010 to September 2011. All study participants have written informed consent and the study was approved by the Ethics Committee of Beijing 302 Hospital.

### Mice

HBV transgenic BALB/c mice were purchased from Transgenic Engineering Lab, Infectious Disease Center (Guangzhou, China), which were generated with a viral DNA construct, pHBV1.3, containing 1.3 copies of the HBV genome. Serum HBV s antigen (HBsAg) and viral DNA, as well as HBc expression in hepatocytes in mice’s liver, were tested positive for all transgenic mice. The HLA-A2.1/Kb transgenic mice (Zhang et al., 2007) were kindly provided by Professor Huang WL (IMCAS, Beijing, China). The HLA-A2.1/Kb mice and HBV transgenic mice were crossed to gain the F1 hybrids of HLA-A2.1/HBV transgenic mice. All F1 hybrids were screened for serum HBsAg by ELISA, viral DNA by real-time PCR, and HLA-A2 by PCR-SSP (PROTRANS, Deutschland) before experimental manipulations.

### Plasmid constructs

The wild-type HBc gene was cloned into pcDNA3.1 (Invitrogen) and the recombinant plasmid was designated pcDNA3.1-HBc. pHBV1.3 containing 1.3 copies of the full-length HBV genomic sequence was maintained in the lab.

### Peptide synthesis

The HBc sequences of genotypes B and C were attained using the protein database from NCBI and a total of 171 HBc sequences of genotype B and 159 HBc sequences of genotype C included. The sequences were compared and served as a basis on peptide synthesis. If the variation rate of a certain amino acid (aa) was more than 10%, a series of peptides associated with this variation would be synthesized. We adopted the overlapping method (8-aa overlap) to synthesize a total of 191 nonapeptides (9-mers) covering HBc1–150 aa (Zhang et al., 2013). All of these peptides were synthesized at Jier Biological (Shanghai, China), and their purity was determined as >95%. The HBc18–27 (FLPSDFFPSV) and HBc82–90 (RELVVSYVN) peptides were used as positive and negative controls, respectively.

### Cell culture and transfection

The 293T cell line was obtained from the ATCC (Manassas, VA, USA) and cultured in Roswell’s Park Memorial Institute medium supplemented with 10% fetal bovine serum. The T2 cell line was kindly provided by Professor BinGao (IMCAS, Beijing, China) and cultured in RPMI 1640 with 10% fetal bovine serum (Gibco BRL, Paisley, UK). The transfection reagent Lipo2000 (Invitrogen, USA) were used in cell transfection according to the instruction provided by the manufacturer. Cells and supernatants were harvested at 24 h, 48 h, and 72 h after transfection, respectively.

### T2 binding assay

T2 cells were used to perform MHC stabilization assays as previously described (Zhou et al., 2006). The binding activity of each peptide was calculated as the fluorescent index (FI), and the FI was determined by: (mean FITC fluorescence with the given peptide − mean FITC fluorescence without peptide) (mean FITC fluorescence without peptide). Peptides regarded as epitopes with high affinity should meet the following criteria: FI ≥1.

### Refolding, protein crystallography, and structure determination

Recombinant proteins of HLA-A*0201 heavy chain and β2m were expressed in *Escherichia coli* (Garboczi et al., 1992.) and the gradual dilution method was performed during refolding process (Liu et al., 2012). Then, Superdex 200 10/300 GL gel filtration chromatography followed by Resource-Q anion-exchanged chromatography (GE Healthcare) was used for the concentration and purification of the soluble portion of the refolded complex. The hanging-drop vapor diffusion method was performed at 18°C to crystallize the purified complexes. At a final concentration of 10 mg/mL in 0.1 mol/L Bis-Tris (pH 6.5) and 25% (*w*/*v*) polyethylene glycol 3350, HLA-A0201/HBc141–149 crystals were obtained. Equipped with an R-AXIS V||++ image-plate detector, Rigaku MicroMax007 rotating-anode X-ray generator was operated at 40 kV and 20 mA (Cu Κα; λ = 1.5418 Å) to collect crystallographic data at 100 K in house. With Protein Data Bank (PDB) entry 1JF1 as the search model, the structure of HLA-A*0201/HBc141–149 was determined by molecular replacement with the program MolRep.

### Immunization of mice

Mice (6–8 weeks old) were immunized i.m. with 50 μg of plasmid pcDNA3.1-HBc or pcDNA3.1 (control) at week 1 and subcutaneously with 50 μg of HBc141–149 peptide bound to 30 μg of heat shock protein gp96 as adjuvant (Li et al., 2011) at weeks 3 and 4, respectively. Mice were sacrificed 1 week after the last immunization. Splenocytes were isolated as previously described (Liu et al., 2009). Each group contained five to seven mice.

### IFN-γ ELISPOT assay

To detect epitope-specific T cells, enzyme-linked immunosorbent spot (ELISPOT) assay was performed according to the manufacturer’s instruction. Briefly, ninety-six well PVDF plates (BD-Pharmigen, San Diego, CA) were precoated overnight at 4°C with the coating Ab and blocked for 1 h at 37°C. Patient PBMCs (2 × 10^5^) or murine splenocytes (10^6^) were added to each well together with 50 μg/mL of peptide and incubated at 37°C for 24–48 h with phytohemagglutinin (PHA)-stimulated T cells as positive controls. Each test was performed at least in triplicate. The spots were counted and analyzed using an ELISPOT Reader (Cellular Technology Ltd, USA).

### Cytotoxicity assay

293T cells were labeled with 2 μmol/L CFSE as target cells after transfection with pHBV1.3 and seeded into a 96-well plate. Then CTLs were added at different ratios: 1:1, 10:1, and 20:1. Plates were incubated for 4–6 h at 37°C, and CFSE positive target cells were stained with propidium iodide (PI) using a Vybrant Apoptosis Assay Kit (Invitrogen, USA). In addition, T2 cells were loaded with 20 μg/mL HBc141–149 peptide at 37°C for 1 h as target cells, and seeded into a 96-well plate. The cytotoxicity assay was performed as described above. Each assay was performed in triplicate.

### Detection of HBsAg and HBeAg by ELISA, and HBV DNA by real-time PCR

ELISAs and real-time PCR for detection of serum HBsAg, HBeAg and viral DNA copies were performed as described (Fan et al., 2013).

### Statistical analysis

Differences between groups were determined using Student’s *t*-test. Pearson’s χ test was used to detect the correlation between variation rate and disease progress in HBV infected patients. Clinical statistical analyses were performed with SPSS version 16.0 software (SPSS Inc., Chicago, Illinois). *P* values <0.05 were considered significant.

## Abbreviations

ACLF: acute on chronic liver failure
AHB: acute hepatitis B
CTL: cytotoxic T lymphocyte
ELISPOT: enzyme-linked immunosorbent spot
FI: fluorescent index
HBc: HBV core protein
HBeAg: HBV e antigen
HBsAg: HBV s antigen
HBV: Hepatitis B virus
SFC: spot-forming cells
